# How Does Risk-Information Communication Affect the Rebound of Online Public Opinion of Public Emergencies in China?

**DOI:** 10.3390/ijerph18157760

**Published:** 2021-07-22

**Authors:** Shan Gao, Ye Zhang, Wenhui Liu

**Affiliations:** 1 School of Public Administration, Central South University, Changsha 410083, China; gs@csu.edu.cn (S.G.); liuwenhui@csu.edu.cn (W.L.); 2 School of Economic Management and Law, University of South China, Hengyang 421001, China

**Keywords:** risk-information communication, public emergencies, public health emergencies, rebound of online public opinion, fsQCA

## Abstract

The rebound of online public opinion is an important driving force in inducing a secondary crisis in the case of public emergencies. Effective risk-information communication is an important means to manage online public opinion regarding emergencies. This paper employs fuzzy-set qualitative comparative analysis to discover which conditions are combined and may result in the rebound of online public opinion. Five conditions were selected: the type of public emergency, messengers, message attributes, audience, and information feedback. The study used a sample of 25 major public emergencies that occurred between 2015 and 2020 in China. The type of public emergency, audience, and information feedback emerged as critical influencing factors. Message attributes promote the rebound of online public opinion regarding public health emergencies, while messengers play a traction role in the rebound of online public opinion on other types of public emergencies. This study extends risk-information communication theory from the perspective of the type of emergency, explores the causes of rebounded online public opinion regarding public emergencies, and provides policies and suggestions for risk-information communication and online public-opinion governance during emergencies.

## 1. Introduction

**Figure 1:**
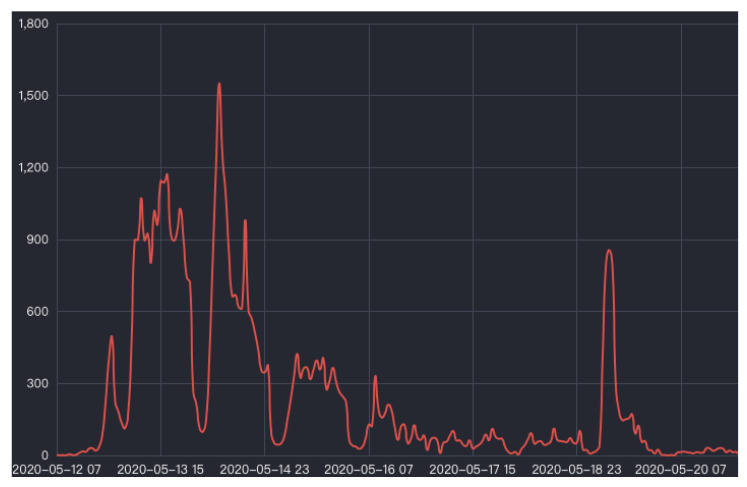
Development trend chart of online public opinion on the “fake milk powder leads to the emergence of big head babies” story. Image comes from Zhiweidata, a website that collects information from the whole network to calculate the communication effect and authoritative indicators of a single event on the Internet, so as to obtain the event influence index and the trend of online public opinion. https://ef.zhiweidata.com/event/b6e0c0e62114908a10034457/trend (accessed on 20 May 2021).

After the occurrence of a public emergency, it is often easy to cause a heated public discussion on network platforms and form online public opinion about the emergency. Research on the evolution life cycle of online public opinion shows that the evolution of online public opinion in emergencies goes through the three periods of formation, climax, and dissipation, or the four periods of gestation, transmission, upsurge, and decline [1,2]. After the outbreak of an event triggers a high level of public opinion, with the disposal of the event and the shift of people’s attention, discussion or the event on a network gradually fades. For example, Ma et al. summarized online public opinion of two fire accidents, and found that the attention of netizens would rapidly decline after exponential growth, with a peak value [3]. Through the analysis of a typhoon event in the Philippines, Ma et al. found that heated online public opinion gradually fades away within 24–48 h after the event [4]. However, with the spread of the hazard, information is released in an untimely manner, and other reasons may make online public opinion regarding emergencies rebound in a certain period of time after a complete public-opinion cycle, and become the catalyst for derivative online public opinion [5,6]. The rebound of online public opinion refers to the situation of it appearing a second or multiple times, with high popularity, after already experiencing a high level of public opinion and decline. As shown in Figure 1 below, the “fake milk powder leads to the emergence of big head babies” incident triggered heated online public opinion within 48 h, and the second heated discussion about the incident, that is, the rebound of online public opinion, occurred in social media on the fifth day after the first had subsided.

Risk communication is a process in which information messengers release risk information to the audience and prevent people’s risk perception from magnifying. Untimely risk-information communication may lead to the widespread spread of false information and negative public emotions in cyberspace, resulting in the continuous attention of netizens to the emergency. As a form of online public opinion about meta-events, the rebound of online public opinion reflects the persistence of people’s attention towards a public emergency. Persistent or repeated high-popularity public opinion provides the time and space for the generation of derivative online public opinion. The occurrence of rebounded online public opinion aggravates the task of public emergency response, damages the image of the government, and even triggers collective panic behavior, posing a threat to social stability [7]. Therefore, it is necessary to discuss the causes of the recurrence of high-popularity online public opinion from the perspective of risk-information communication.

Existing studies of online public opinion of public emergencies mainly focus on the generation of high-heat online public opinion [8,9,10], taking this as the result variable, discussing the influence of information factors, government response, and other factors on high-heat online public opinion [11,12], and paying little attention to whether emergencies produce secondary or multiple discussions. Why does the high-heat online public opinion of some public emergencies gradually fade within a certain period of time, while some public emergencies cause repeated high-heat online public opinion? This may be related to the continuous spread of risks. With the expansion of the scope of hazards during an event, people’s risk perception continuously increases, which has aroused widespread attention [13]. After the event, low-efficiency risk-information communication factors, such as the release of false news, failure to promptly meet the public’s information demands and distrust of messengers, may also lead to continuous panic and worry among the public [14,15,16], and induce repeated high public opinion, generating the rebound of network public opinion. Some studies have also confirmed the relationship between the type of emergency and online public opinion. As pointed out by Malik et al. in the area of risk communication, it is significant to recognize that not all outbreaks are the same and that targeted communication strategies are needed for different outbreaks [17].

In this context, this paper elaborates the role of the type of emergency and risk-information communication factors, after the occurrence of an emergency, in the rebound of network public opinion, so as to better carry out measures for the governance of online public opinion of emergencies. This paper focuses on an evolutionary form of online public opinion regarding public emergencies, namely, the rebound of online public opinion, that is, the re-emergence of high-heat public opinion after it has faded away. The study provides insight into the factors that can influence the rebound of online public opinion by investigating the role of the type of incident and risk-information communication after an emergency.

The remainder of the paper is organized as follows. In Section 2, the theoretical framework is presented. In Section 3, key elements of the rebound of online public opinion and the dataset are described, and the methodological aspects are explained. Section 4 focuses on data analysis and results, while Section 5 presents the conclusion, policy implications, and research limitations.

## 2. Theoretical Framework

**Figure 2:**
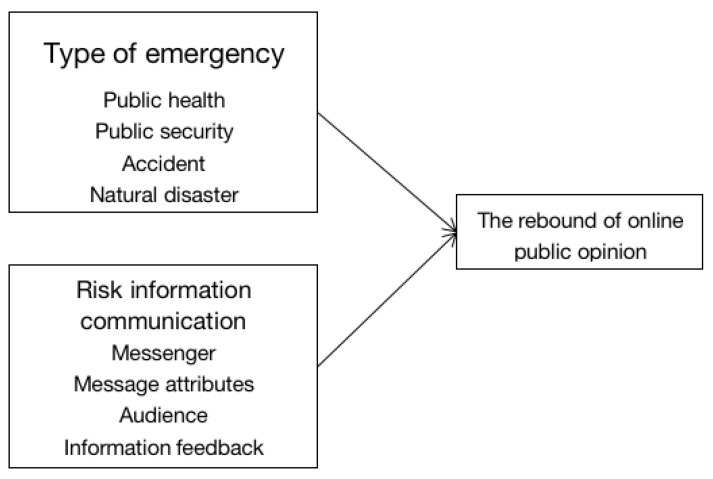
Influence-factor model of the rebound of online public opinion in emergencies.

Communication refers to a social relationship between individuals in which messages emanating from one member of the relationship may enable another member to reduce their “uncertainty” [18]. The message must be based on language that refers to various possible discriminations that an individual can make among features of their environment. Schramm pointed out that risk communication is the process of information sharing with others with three basic elements: messengers, message attributes, and audiences [19]. Lasswell summarized the process of information transmission from transmitter to receiver through the media into five elements, namely, communicator, information, media, audience, and feedback [20]. With the development of risk-communication research, attention has been paid to the interaction between information transmitter and information receiver in risk communication. The American Committee on Risk Cognition and Risk Communication proposed that risk communication is a process of interactive debate on risk information among multiple subjects [21]. Risk-information communication on the network is a process in which multiple subjects, such as the government, experts, opinion leaders, and the general public, discuss and exchange views on events in cyberspace [22]. In risk-information communication after the occurrence of a public emergency, a public-emergency meta-event is the starting point of online public opinion and the initial carrier of online public-opinion topics. The meta-event causes different degrees of online discussion and public attention due to different impact scale and damage scope [9]. On this basis, this paper divides the factors that affect the rebound of online public opinion on emergencies into five categories: the type of emergency, messengers, message attributes, audience and information feedback. Figure 2 is the theoretical framework of this paper.

First, we deal with the type of public emergency. The impact, scope and scale of emergencies affect the public’s risk perception [23], thus affecting the public’s attention to risk [24]. Saleem believes that the duration and severity of disasters affect people’s participation in discussion on Twitter [25]. According to analysis of the Three Gorges Project fire accident by Jiang et al., meta-event information impacts online public opinion [26]. Dong et al., through their study of online public opinion of natural disasters found that the evolution trend of online public opinion was different even for similar natural disasters due to different properties such as duration and damage degree [27]. Han indicated that the change in online public opinion during COVID-19 was synchronized with the development trend of the pandemic [28]. The development characteristics of online public opinion of emergencies are closely related to the change characteristics of the emergencies themselves. Generally, public health emergencies may affect the living environment, and threaten people’s lives and health, having lasting negative effects. Therefore, compared with other types of event, public health emergencies are more likely to cause a rebound of online public opinion. On this basis, the first proposition is set: the type of emergency influences the rebound of online public opinion of public emergencies.

Second come messengers, which refers to the source of risk information, namely, individuals, groups, and organizations that purposefully communicate with risk. In a public emergency, governments, experts, opinion leaders, and the general public are common sources of risk information. Pourebrahim et al. found that news organizations, political figures, disaster-related institutions, and organizations were the main sources of information that influenced online public opinion through their interconnection with Twitter users during Hurricane Sandy [29]. Ma et al. divided network information sources for large-scale fire accidents into netizens, the media, political parties, opinion leaders, and the government [3]. With the development of social media, trust plays an important role in almost all risk fields, such as nuclear power and climate change [30,31,32], and trust in information sources affect people’s choice of risk information and the interpretation of information released by these information sources [33,34]. The use of media with a high audience rating but low credibility to publish risk information triggers a high degree of online public opinion [35,36]. On the basis of the above analysis, the second proposition is: messengers influence the rebound of online public opinion of public emergencies.

Third are message attributes, which refers to the characteristics of the language used by the messenger to express the message, including the authenticity and the use of the framework of language. The expression of risk information can redefine risk perception and change people’s definition of risk [37]. Peters et al. found that a lack of information on the efficacy of measures to inform people to take to reduce their exposure to risks may lead people to ignore risks [38]. Untested, false information that appears to be true could have the opposite effect on risk communication and amplify people’s perception of risk [39]. Depoux indicated that, during COVID-19, the spread speed of false information in the network was much faster than the spread speed of the virus, and was more likely to cause widespread network panic [40]. As a kind of false information, rumors attract wide attention and high public opinion due to their wide spread. Lan et al. and Liu indicated that online rumors prompt the public to generate more online public-opinion topics about the event, the amount of online public opinion would continue to increase, and the upper limit of this increase was difficult to predict [41,42]. On the basis of the above analysis, the third proposition is defined: message attributes influence the rebound of public opinion of public emergencies.

Fourth is the audience, which is the variety of intended message receivers who engage in risk conversations in a variety of ways. Any risk communication message is filtered through the receiving audience’s own selective lenses, with risk perceptions continuing to dominate the literature. Understanding the risk perception of the audience is an important prerequisite for carrying out targeted risk communication [43]. The public’s emotions reflect the public’s risk perception [44], and fear and worry can highly predict people’s risk perception [45]. Under the perception of high risk, people express their emotions or worries through the Internet and form online public opinions [46]. Wang et al. proved a correlation between public panic and online public-opinion crises in emergencies through multiple case studies [47]. Li and Peng et al. used the sentiment index to measure the public-opinion index regarding public emergencies. Their research proved that the changes in netizens’ emotions in public emergencies reflected changes in the online public opinion of the event [48,49]. On the other hand, the media can promptly channel the negative emotions of the public and change the direction of risk perception, which can play a role in controlling the spread of online public opinion of events [50]. Therefore, the fourth proposition is that the negative emotions of audiences influence the rebound of online public opinion of public emergencies.

Fifth is information feedback, which is a process of dialogue between messengers and audience, promptly satisfying the information demands of the audience and giving feedback. The development of social media has provided a new channel for information feedback, and messengers can deliver information more quickly to a wider and more targeted audience [51]. Information receivers are not passive. They expect to promptly receive feedback after they express their information demands through information channels. Liu’s analysis of the Liangshan fire incident found that one-way risk-information communication was an urgent risk communication problem, which was an influencing factor on online public opinion [7]. Zhao et al. pointed out that a lack of information and untimely information feedback may provide a space for the generation of online rumors and draw much of the attention of netizens [52]. Xiang, through a case study, found that the prompt disclosure of public-emergency information on government microblogs could prevent the spread of the negative impact of an event on microblog platforms [53]. Therefore, prompt information feedback is an important factor that affects the generation of online public opinion. Therefore, the fifth proposition is that information feedback influences the rebound of online public opinion of public emergencies.

## 3. Methodology

### 3.1. Sample and Data

Social media are increasingly used to obtain information about public opinion, sentiment, and key factors related to various objects of study. For example, Reyes-Menendez analyzed Twitter users’ emotional experiences by analyzing tweet hashtags [54,55]. Liu et al. used text information in microblogs to determine people’s opinions and attitudes towards an emergency [7]. Weibo and WeChat are the most widely used social platforms in China. The summary of Weibo and WeChat users’ opinions can reflect the online public opinion of a certain event. Zhiweidata is an aggregation platform of social hot spots on the Internet. Through the comprehensive evaluation and calculation of the communication effect of an event on the three platforms of Weibo, WeChat, and online media, the influence index of an event is obtained. The greater the value, the higher the attention of an event on the Internet. In order to ensure the typicality of case selection, 25 major emergencies were selected from the Zhiweidata webpage with an influence index of more than 70 that occurred in 2015–2020 (case samples are detailed in Appendix A Table A1).

### 3.2. Method: fsQCA

Qualitative comparative analysis (QCA) is a research method to solve complex social problems induced by multiple causes. It solves the problem of multiple complex concurrent causality, and focuses on how a number of different condition variables lead to results in combination. QCA is a combination of qualitative and quantitative research methods, and aims to explore the common characteristics of cases by discussing the subordinate relationship between sets [56]. fsQCA is a qualitative comparative analysis method based on fuzzy sets. According to the degree of subordination of variables, the subordinate degree of variables is defined as between non-subordination (0) and full subordination (1) [57]. Through the analysis of necessary and sufficient conditions for the generation of the result, QCA finds a variety of combination relations leading to the result. It is also a method of empirical research that tests results, assumes that multiple causal paths may coexist to obtain the results, and interprets the results in different ways. 

QCA is applicable to this study. On the one hand, QCA can verify the combination relationship between multiple factors that produce a certain result. The occurrence and development of online public opinion are the result of the joint action of multiple factors, such as the flood of false information, and the widespread spread of negative emotions such as panic and fear; all play an important role in the development and occurrence of online public opinion. On the other hand, QCA’s causality analysis of small and medium-sized sample cases is more advantageous than regression analysis. In this study, 25 cases were selected to explore the influencing factors of online public-opinion rebound, which is suitable for QCA. In addition, factors affecting the rebound of online public opinion are often difficult to distinguish by full or incomplete subordination, so it is necessary to classify the factors by fuzzy subordination degree, which is suitable for fsQCA.

The specific application steps of QCA are as follows. (1) Establish the result variables and condition variables of the study; the outcome variable of this paper is whether the online public opinion of an emergency has rebounded, and the antecedent conditional variable is the type of emergency and the risk communication factor. (2) Establish a truth table to list all logical combinations leading to the existence or nonexistence of the result. (3) Import the truth table into fsQCA software for calculation, and analyze the necessary and sufficient conditions for generation of the results.

### 3.3. Variable Interpretation and Assignment

To perform the analysis, the outcome must first be defined. In this case, the outcome represents the rebound of online public opinion; the presence of the outcome means that the rebound of online public opinion is generated, while the absence of the outcome means that the rebound of online public opinion is not generated. In addition to the outcome, the conditions that can influence the result must be selected. According to the theoretical framework described above, five antecedent conditions were set: type of public emergency (TP), messenger (ME), message attributes (MA), audience (NE), and information feedback (IF). Table 1 provides a description and codification of the outcome and conditions.

All condition variables except the messenger were in crisp condition (0 or 1). If the condition was satisfied, the value was assigned to 1; if not, the value was assigned to 0. The value of the messenger variable was divided into four categories according to the mean anchor point method. The value was assigned according to the type of variable to which the case belonged: 1 for central news media, 0.67 for local news media, 0.33 for other news media, and 0 for social media.

### 3.4. Truth-Table Construction

After the assignment of variables, regression cases are required to code and summarize each case in order to construct the truth table. The truth table lists possible combinations of causal conditions after removing contradictory configurations and identifying the logical remainder. The truth table constructed in this paper on the basis of the result variables and antecedent variables is shown in Table 2 below.

## 4. Data Analysis and Results

### 4.1. Single-Factor Necessity Analysis: Motivation for the Rebound of Online Public Opinion

The purpose of single-factor necessity analysis is to explore the conditions that must occur for the presence of the outcome or the absence of the outcome. The Necessary Condition command in fsQCA software can accomplish this step. Table 3 shows the results of single-factor necessary-condition analysis, according to Schneider and Wagemann [58]. If the consistency score of a factor is greater than 0.9, it means that this factor is a necessary condition for the presence or absence of the outcome. The consistency coefficient was greater than 0.8, indicating that this index was a sufficient condition for the occurrence of the result. Coverage rate judges the explanatory strength of antecedent conditions on the result variable, and “~” means “not”, i.e., the opposite value.

For the result of the presence of rebounded online public opinion, the consistency score of ~IF was higher than 0.9, which indicated that information feedback was a necessary condition for the generation of rebounded network public opinion. The consistency of NE was greater than 0.8, indicating that negative sentiment was a sufficient condition for the rebound of online public opinion. Single-factor necessity analysis showed that the factor that could independently trigger the rebound of network public opinion was information feedback. Negative emotions, though not necessary, play an important role in the outcome.

For the result of the absence of rebounded online public opinion, the consistency scores of ~TP and ~NE were higher than 0.9, indicating that both are necessary conditions for non-rebounded online public opinion. The above analysis shows that the important premise for emergency online public opinion not to rebound is that the event is not a public health emergency and does not cause negative emotions in the audience. 

### 4.2. Conditional Combination Analysis: Generated Path of the Rebound of Online Public Opinion

The purpose of conditional combination analysis is to find important influencing factors or combination paths that influence the presence or absence of an outcome. To perform analysis, the consistency cut-off for the presence or absence of results was set to 0.8. fsQCA software was used to calculate the combination of conditions for the presence or absence of the rebound of online public opinion, and the three combination schemes of complex solution, intermediate solution, and parsimonious solution were obtained. A complex solution is a result obtained by setting parameters completely according to the variables, which is often used as the preferred scheme for QCA [59]. 

As shown in Table 4 below, there were three combined paths to generate the result of the rebound of public opinion, namely 1, 2, and 3. The solution coverage of condition combinations with the presence of rebounded online public opinion was 0.821539, indicating that these configurations could explain more than 82% of the cases. Among the three combined paths, the original coverage rate of two paths was higher than the unique coverage rate, indicating that there were support cases in line with multiple causal paths. Through the integration of three paths, the paths of online public-opinion rebound in emergencies could be simplified into two: (1) Out = TP * (MA + NE) * ~IF; (2) OUT = ~TP * ME * NE * ~IF. The simplified paths show that untimely information feedback is a necessary condition for the rebound of online public opinion, and the message attributes and the negative emotions of the audience also play an important role in promoting the rebound of online public opinion of public health emergencies. Messengers do not play a core role in the rebound of public opinion of public health emergencies, but play an important role in the rebound of public opinion of other types of public emergencies.

As shown in Table 5 below, the solution coverage of conditional combinations without rebounded online public opinion was 0.779167, indicating that these configurations could explain more than 77% of the cases. There were four combined paths to generate the result of the non-rebound of online public opinion, namely, 4, 5, 6, and 7. Since the raw coverage of combination 7 was 0.0558333, indicating that it could only explain 5% of the cases, the combination’s explanatory power was low, while the coverage rates of other combinations were all higher than 0.2, indicating that they could explain more than 20% of the cases; thus, combination 7 was excluded from the analytical scope, and configurations 4–6 were selected for analysis. Through the integration of paths 4–6, the formula of non-rebound online public opinion in public emergencies could be simplified as ~OUT = ~ TP * (~MA + IF) * ~NE. The simplified formula shows that a nonpublic health emergency and the non-negative emotions of the audience were the core elements of the condition combination, and message attributes and information feedback played a driving role.

## 5. Discussion

By using the fsQCA method, this article explored the influence of factors and their combination on the rebound of online public opinion of public emergencies. Analysis mainly focused on the type of public emergency and risk-information communication factors after the occurrence of public emergencies.

The results of single-factor research showed that the consistency of information feedback was higher than 0.9, which is a necessary condition for the rebound of online public opinion. Proposition 5 was confirmed. This research conclusion is consistent with Chen’s opinion, i.e., the more promptly that public-emergency information is fed back, the less likely it is to induce secondary public opinion [60]. 

The type of emergency (not public health emergencies) and the absence of negative emotions in the audience are necessary conditions for the non-rebound of online public opinion in public emergencies. The above results demonstrate the correctness of propositions 1 and 4, respectively. This conclusion is different from that proposed by Li et al. [10], namely, the more harmful the emergency, the more likely it is to generate a high amount of public opinion. Due to the timely handling of these emergencies, and the government formulating effective policies for post-disposal, there was often no rebound of public opinion in earthquake and fire emergencies, although they were very harmful. However, almost each individual involved in the public health emergency focused on it for a long time, making it easy to cause repeated high-heat public opinion, such as with biological vaccines and fake milk-powder events, when people were very concerned about whether they had been harmed and expressed anger over the government’s lack of timely supervision, inducing the rebound of online public opinion. 

The results of condition combination analysis showed that the rebound path of online public opinion of public health emergencies is different from that for other types of public health emergencies. In public health emergencies, message attributes play an important role in the rebound of online public opinion, while in other types of public emergencies messengers play an important role. The above results partly prove the applicability of Propositions 2 and 3. For example, during COVID-19, rumors constantly occupied the page of hot search on Weibo, making the amount of online public opinion on the epidemic situation continue to rise. The reason for the online public opinion rebound of other public emergencies, such as the bus incident in Anshun, Guizhou Province, is that official media such as people.com.cn reported the cause of the incident, which attracted extensive social attention. Due to the longer duration of public health emergencies, the degree of harm is larger, and development trends and disposal information receive more attention. During public health emergencies, people pay more attention to information related to the identification, transmission and prevention of diseases [61]. Furthermore, in order to draw more attention, some network users often post false information, and widely spreading false information is easy to arouse people’s negative emotions, then inducing the rebound of online public opinion. 

According to the path of online public-opinion rebound in other types of public emergency, messengers have an important influence on social media [62]. In other types of public emergency, because the cause of the emergency or the disposal information of the emergency is not released in time, once the official media report the relevant information, this may cause widespread anger and fear, and this arouses a wide range of discussions and sets off public opinion once again, e.g., the accident in which buses fell into the river in Chongqing and Guizhou Province. Five to seven days later, the cause of the accident was released, which triggered condemnation and anger on the Internet and led to the fermentation of public opinion once again.

## 6. Conclusions

This study focused on the factors that trigger the rebound of online public opinion in public emergencies. Results showed that three of the five factors had independent influence on the results, that is, the type of public emergency, negative emotions of the audience, and the timeliness of information feedback. Message attributes play an important role in the online public-opinion rebound of public health emergencies, while messengers play an important role in the online public-opinion rebound of other types of public emergencies.

The aforementioned findings also significantly contribute to management practices. First is the timely information feedback during emergencies. The main reason for the lack of risk communication is that the local government is slow to leak information [63]. For a public health emergency with widely spreading hazards, people pay more attention to the situation and the government’s handling. Therefore, the scope and extent of the harm of the emergency should be tracked and relevant interest groups should be informed in a timely manner. For public emergencies with non-spreading hazards, people are more concerned about the cause. Therefore, a timely investigation of the cause of an accident and its timely disclosure are very important to prevent the occurrence of repeated high-popularity public discussions.

Second comes reducing the traction of message attributes in the rebound of online public opinion in public health emergencies. Communication transparency, such as completeness, and clarity of information, were critical in communication from public health authorities [64]. On the one hand, a false-information monitoring mechanism for public health emergencies should be established to monitor fast-spreading information and identify false information in the network in a timely manner. On the other hand, digital technologies such as big data and artificial intelligence should be used to establish a real-time information-update mechanism to respond to and correct false information in a timely manner.

Lastly, is giving full play to emotional guidance from official media in other types of public emergencies. The official media are publishers of authoritative information, and should release positive news in a timely manner to guide public emotions towards positive ones. A cooperation mechanism with opinion leaders should be established to guide opinion leaders to release positive information when negative information is released by the authoritative media, so as to form a neutral opinion. Positive language should also be chosen to release emergency information, in order to reduce the amplification effect of negative language on negative emotions.

The main contribution of this paper is based on the effect of risk-information communication on online public-opinion rebound, joining variable event types, and discussing the different paths of public-health and other types of emergency on online public-opinion rebound. It provides theoretical and practical thinking for the online public-opinion governance of public emergencies, especially public health emergencies, and enriches the theoretical scope of risk-information communication. This paper focuses on online public-opinion rebound, which is a form of public opinion. Public-opinion rebound has received less attention in academia. This research has also expanded the scope of online public-opinion research to a certain extent. 

There are some limitations in this study. First, the data were mostly secondary. Although carefully screened, there may still have been deviation in data selection. Second, fuzzy qualitative analysis is comparative analysis among multiple cases, so it is difficult to analyze the specific circumstances of individual cases. Lastly, the rebound of online public opinion may also be related to such factors as the information environment, the government’s disposal, from the perspective of risk information communication, and failing to take into account other factors influencing the rebound of online public opinion. Future studies could discuss the generating mechanism of online public opinion regarding public emergencies from the perspective of government intervention.

## Figures and Tables

**Table 1:** Settings of results and condition variables.

Variable Source	Coding Standards	Codification
Type of the public emergency	The incident was considered a public-health incident.	1
	The incident was not considered a public-health incident.	0
Messengers	The information on the highest point of online public opinion for the first time was released by the central news organizations, such as People’s Daily and China News Network.	1
	The information on the highest point of online public opinion for the first time was released by local news units, such as local government websites and Rednet.	0.67
	Information on the highest point of online public opinion for the first time was released by network media, such as The Paper, Sina, and Net Ease.	0.33
	Information on the highest point of online public opinion for the first time was released by We Media, such as Internet Big Vs and personal accounts.	0
Message attributes	There was high-heat misinformation about the emergency in the network.	1
	There was no high-heat misinformation about the emergency in the network.	0
Audiences	The incident induced widespread negative public sentiment in the network.	1
	The incident did not induce widespread negative public sentiment in the network.	0
Information feedback	Response to public demands within 24 h after the first peak of public opinion.	1
	No response to public demands within 24 h after the first peak of public opinion.	0
Results of variables	Emergencies triggered resilient online public opinion.	1
	Emergencies did not trigger resilient online public opinion.	0

**Table 2:** Truth table.

TP	ME	MA	NE	IF	OUT
0	1	0	1	0	1
0	1	0	0	1	0
0	1	1	0	0	1
0	0.33	0	1	0	0
0	1	0	0	0	0
1	1	1	1	0	1
0	1	1	0	0	0
0	0.33	1	0	1	0
1	1	1	0	0	1
1	0.33	0	1	0	1
0	1	1	1	0	1
0	0.33	1	0	1	0
1	0.33	0	1	0	1
0	0.33	1	0	1	0
1	1	0	0	1	0
1	0.33	1	1	0	1
0	0.33	0	0	1	0
1	0.33	1	1	0	1

**Table 3:** Single-factor necessity analysis.

Conditions	Presence		Absence	
	Consistency	Coverage	Consistency	Coverage
TP	0.615385	0.888889	0.083333	0.111111
~TP	0.384615	0.312500	0.916667	0.687500
ME	0.793846	0.544017	0.720833	0.455983
~ME	0.206154	0.444444	0.279167	0.555556
MA	0.615385	0.666667	0.333333	0.333333
~MA	0.384615	0.384615	0.666667	0.615385
NE	0.846154	0.916667	0.083333	0.083333
~NE	0.153846	0.153846	0.916667	0.846154
IF	0.000000	0.000000	0.666667	1.000000
~IF	1.000000	0.764706	0.333333	0.235294

**Table 4:** Conditional combination path analysis results of the rebound of online public opinion in public emergencies.

No.	Configuration	Raw Coverage	Unique Coverage	Consistency
1	TP * ~ME * NE * ~IF	0.206154	0.155385	1
2	~TP * ME * NE * ~IF	0.307692	0.307692	0.923788
3	TP * ME * MA * ~IF	0.358462	0.307692	1
Solution coverage: 0.821539; solution consistency: 0.970027.				

Note: *, “and”; ~, “not”, which is the opposite value.

**Table 5:** Results of conditional combination path analysis on online public opinion of public emergencies not rebounding.

No.	Configuration	Raw Coverage	Unique Coverage	Consistency
4	~TP * ME * ~MA * ~NE	0.444167	0.166667	1
5	ME * ~MA * ~NE * IF	0.360833	0.0833333	1
6	~TP * ~ME * ~NE * IF	0.223333	0.195833	1
7	~TP * ~ME * ~MA * NE * ~IF	0.0558333	0.0558333	1
Solution coverage: 0.779167; solution consistency: 1.				

Note: “*” means “and”, and “~” means “not”, which is the opposite value.

## Appendix A

**Table A1:** 25 public emergencies that occurred in China between 2015 and 2020.

No.	Name of Emergency	Year	The Accident Types
1	Bus plunged into a reservoir in Anshun, Guizhou	2020	Public security
2	Oil-tanker explosion in Wenling, Zhejiang	2020	Accident
3	Designated quarantine hotel collapsed in Quanzhou, Fujian	2020	Accident
4	Forest fire broke out in Xichang, Sichuan	2020	Natural disasters
5	A hotel collapsed in Linfen, Shanxi	2020	Accident
6	Tangshan 5.1 earthquake	2020	Natural disasters
7	“Fake milk powder” led to the emergence of big head babies in Hunan	2020	Public health
8	The outbreak of the novel coronavirus pneumonia occurred in Wuhan and other places	2020	Public health
9	No. 7 Experimental School in Chengdu gave students moldy food	2019	Public health
10	A viaduct collapsed in Wuxi	2019	Accident
11	A 6.0 magnitude earthquake hit Changning, Yibin, Sichuan	2019	Natural disasters
12	An explosion at the Xiangshui chemical plant in Yancheng, Jiangsu province	2019	Accident
13	Forest fire breaks out in Liangshan, Sichuan	2019	Natural disasters
14	Bus falls into a river in Wanzhou, Chongqing	2018	Accident
15	Supertyphoon Mangkhut caused landfall	2018	Natural disasters
16	Flood discharge incident in Shouguang, Shandong	2018	Natural disasters
17	Swine, cattle, and sheep outbreaks occurred in many parts of the country	2018	Public health
18	Changsheng biological-vaccine fraud incident	2018	Public health
19	A 7.0 magnitude earthquake hit Jiuzhaigou	2017	Natural disasters
20	Typhoon Hato	2017	Natural disasters
21	More than 100 people were buried when a mountain collapsed in Diaxi, Sichuan	2017	Accident
22	MERS invades Guangdong	2015	Public health
23	Landslide in Shenzhen	2015	Public security
24	Landslide in Lishui, Zhejiang province	2015	Natural disasters
25	Tianjin port explosion	2015	Accident

## References

[1] Wang X., Zhao D., Junwei W. (2015). Empirical research on modeling of online public opinion propagation in a mobile environment: An example focusing on the topic of “Ebola”. J. China Soc. Sci. Tech. Inf..

[2] Chuanming A.L.D.T.Y., Gang Z.L.L. (2016). Microblogging topic evolution pattern and timing trends of public health emergencies: Taking Ebola microblogging on Twitter and Weibo for example. Inf. Doc. Serv..

[3] Ma Y., Shu X., Shen S., Song J., Li G., Liu Q. (2014). Study on network public opinion dissemination and coping strategies in large fire disasters. Procedia Eng..

[4] Ma X., Liu W., Zhou X., Qin C., Chen Y., Xiang Y., Zhang X., Zhao M. (2020). Evolution of online public opinion during meteorological disasters. Environ. Hazards.

[5] Zhu H., Hu B. (2018). Impact of information on public opinion reversal—An agent based model. Phys. A Stat. Mech. Its Appl..

[6] Jiang G., Li S., Li M. (2020). Dynamic rumor spreading of public opinion reversal on Weibo based on a two-stage SPNR model. Phys. A Stat. Mech. Its Appl..

[7] Liu Y., Zhu J., Shao X., Adusumilli N.C., Wang F. (2021). Diffusion patterns in disaster-induced internet public opinion: Based on a Sina Weibo online discussion about the “Liangshan Fire” in China. Environ. Hazards.

[8] Li M., Cao H. (2020). Research on the generating mechanism of online public opinions of emergent events from the perspective of information ecology—Based on the qualitative comparison and analysis of 40 emergent events with clear set. Inf. Sci..

[9] Gao S., Zhang G., Sun X., Yang F. (2019). The internal logic of network public opinion crisis of secondary public crisis—A qualitative comparative analysis of fuzzy set based on 40 cases. Public Adm. Rev..

[10] Li W., Gao G. (2020). Research on the generating mechanism of online public opinion heat in public emergencies—Qualitative comparative analysis of fuzzy sets based on 48 cases (fsQCA). Intell. Mag..

[11] Yu L., Li L., Tang L. (2017). What can mass media do to control public panic in accidents of hazardous chemical leakage into rivers? A multi-agent-based online opinion dissemination model. J. Clean. Prod..

[12] Zhang W., Wang M., Zhu Y. (2020). Does government information release really matter in regulating contagion-evolution of negative emotion during public emergencies? From the perspective of cognitive big data analytics. Int. J. Inf. Manag..

[13] Grimm A., Hulse L., Preiss M., Schmidt S. (2012). Post- and peritraumatic stress in disaster survivors: An explorative study about the influence of individual and event characteristics across different types of disasters. Eur. J. Psychotraumatol..

[14] Ding X., Zhang X., Fan R., Xu Q., Hunt K., Zhuang J. (2021). Rumor recognition behavior of social media users in emergencies. J. Manag. Sci. Eng..

[15] Fernández-Torres M.J., Almansa-Martínez A., Chamizo-Sánchez R. (2021). Infodemic and fake news in Spain during the COVID-19 pandemic. Int. J. Environ. Res. Public Health.

[16] Sopory P., Novak J.M., Day A.M., Eckert S., Wilkins L., Padgett D.R., Noyes J.P., Allen T., Alexander N., Vanderford M.L. (2021). Trust and public health emergency events: A mixed-methods systematic review. Disaster Med. Public Health.

[17] Malik A., Khan M.L., Quan-Haase A. (2021). Public health agencies outreach through Instagram during the COVID-19 pandemic: Crisis and emergency risk communication perspective. Int. J. Disaster Risk Reduct..

[18] Carroll J.B. (1958). Chapter I: Communication theory, linguistics, and psycholinguistics. Rev. Educ. Res..

[19] Schramm W. (1962). Mass communication. Annu. Rev. Psychol..

[20] Lasswell H.D., Bryson L. (1948). The communication of ideas. The Structure and Function of Communication in Society.

[21] National Research Council (US) Committee on Risk Perception and Communication (1989). Improving Risk Communication.

[22] Chen J. (2020). Risk Communication in cyberspace: A brief review of the information-processing and mental models approaches. Curr. Opin. Psychol..

[23] Ho M.-C., Shaw D., Lin S., Chiu Y.-C. (2008). How do disaster characteristics influence risk perception?. Risk Anal..

[24] Geng S., Zhou Q., Li M., Song D., Wen Y. (2021). Spatial–temporal differences in disaster perception and response among new media users and the influence factors: A case study of the Shouguang flood in Shandong province. Nat. Hazards.

[25] Saleem H.M., Xu Y., Ruths D. (2014). Effects of disaster characteristics on Twitter event signature. Procedia Eng..

[26] Jiang H., Qiang M., Lin P. (2016). Assessment of online public opinions on large infrastructure projects: A case study of the three gorges project in China. Environ. Impact Assess. Rev..

[27] Dong Z.S., Meng L., Christenson L., Fulton L. (2021). Social media information sharing for natural disaster response. Nat. Hazards.

[28] Han X., Wang J., Zhang M., Wang X. (2020). Using social media to mine and analyze public opinion related to COVID-19 in China. Int. J. Environ. Res. Public Health.

[29] Pourebrahim N., Sultana S., Edwards J., Gochanour A., Mohanty S. (2019). Understanding communication dynamics on Twitter during natural disasters: A case study of Hurricane Sandy. Int. J. Disaster Risk Reduct..

[30] Smith E.K., Mayer A. (2018). A social trap for the climate? Collective action, trust and climate change risk perception in 35 countries. Glob. Environ. Chang..

[31] Saleh R., Bearth A., Siegrist M. (2019). “Chemophobia” today: Consumers’ knowledge and perceptions of chemicals. Risk Anal..

[32] Blair R.A., Morse B.S., Tsai L.L. (2017). Public health and public trust: Survey evidence from the Ebola virus disease epidemic in Liberia. Soc. Sci. Med..

[33] Fischhoff B., Wong-Parodi G., Garfin D.R., Holman E.A., Silver R.C. (2018). Public understanding of Ebola risks: Mastering an unfamiliar threat. Risk Anal..

[34] Trumbo C.W., McComas K.A. (2003). The function of credibility in information processing for risk perception. Risk Anal..

[35] Entradas M. (2021). In Science We Trust: The Effects of Information Sources on COVID-19 Risk Perceptions. Health Commun..

[36] Löfstedt R., Way D., Bouder F., Evensen D. (2016). Transparency of medicines data and safety issues—A European/US study of doctors’ opinions: What does the evidence show?. J. Risk Res..

[37] Boholm Å. (2009). Speaking of risk: Matters of context. Environ. Commun..

[38] Peters G.-J.Y., Ruiter R.A.C., Kok G. (2013). Threatening communication: A critical re-analysis and a revised meta-analytic test of fear appeal theory. Health Psychol. Rev..

[39] Hart P.S., Nisbet E.C. (2012). Boomerang effects in science communication: How motivated reasoning and identity cues amplify opinion polarization about climate mitigation policies. Commun. Res..

[40] Depoux A., Martin S., Karafillakis E., Preet R., Wilder-Smith A., Larson H. (2020). The pandemic of social media panic travels faster than the COVID-19 outbreak. J. Travel Med..

[41] Lan Y., Lian Z., Zeng R., Zhu D., Xia Y., Liu M., Zhang P. (2020). A statistical model of the impact of online rumors on the information quantity of online public opinion. Phys. A Stat. Mech. Its Appl..

[42] Liu Q., Li T., Sun M. (2017). The analysis of an SEIR rumor propagation model on heterogeneous network. Phys. A Stat. Mech. Its Appl..

[43] Siegrist M., Árvai J. (2020). Risk perception: Reflections on 40 years of research. Risk Anal..

[44] Burns W.J., Peters E., Slovic P. (2012). Risk perception and the economic crisis: A longitudinal study of the trajectory of perceived risk. Risk Anal..

[45] Skagerlund K., Forsblad M., Slovic P., Västfjäll D. (2020). The affect heuristic and risk perception—Stability across elicitation methods and individual cognitive abilities. Front. Psychol.

[46] González-Bailón S., Paltoglou G. (2015). Signals of public opinion in online communication: A comparison of methods and data sources. ANNALS Am. Acad. Political Soc. Sci..

[47] Wang Z., Liu X., Zhang S. (2019). A new decision method for public opinion crisis with the intervention of risk perception of the public. Complexity.

[48] LI Y., Sun L., Li S., Zhou Y. (2020). Temporal and spatial distribution of netizens’ risk perception in public risk events: Empirical experience from H7N9. Intell. Mag..

[49] Peng Z., Huang H., Wu H., Xie Q. (2020). Big data analysis of COVID-19 emergency prevention and control in the early stage of COVID-19 epidemic. Gov. Res..

[50] Arafat S.M.Y., Kar S.K., Menon V., Kaliamoorthy C., Mukherjee S., Alradie-Mohamed A., Sharma P., Marthoenis M., Kabir R. (2020). Panic buying: An insight from the content analysis of media reports during COVID-19 pandemic. Neurol. Psychiatry Brain Res..

[51] Sutton J., VEIL S.R. (2017). Risk Communication and Social Media. Risk Conundrums: Solving Unsolvable Problems.

[52] Zhao L., Wang Q., Cheng J., Zhang D., Ma T., Chen Y., Wang J. (2012). The impact of authorities’ media and rumor dissemination on the evolution of emergency. Phys. A Stat. Mech. Its Appl..

[53] Xiang L. (2019). Study on the application of government affairs micro-blog in the disclosure of government emergency information in China. Disaster Prev. Manag. Int. J..

[54] Reyes-Menendez A., Saura J.R., Alvarez-Alonso C. (2018). Understanding #WorldEnvironmentDay user opinions in Twitter: A topic-based sentiment analysis approach. Int. J. Environ. Res. Public Health.

[55] Reyes-Menendez A., Saura J.R., Palos-Sanchez P. (2020). Identifying key performance indicators for marketing strategies in mobile applications: A systematic literature review. Int. J. Electron. Mark. Retail..

[56] Ragin C.C. (2008). Redesigning Social Inquiry: Fuzzy Sets and Beyond.

[57] Fiss P.C. (2007). A set-theoretic approach to organizational configurations. AMR.

[58] Schneider C.Q., Wagemann C. (2010). Standards of good practice in Qualitative Comparative Analysis (QCA) and fuzzy-sets. Comp. Sociol..

[59] Huarng K.-H., Yu T.H.-K., Rodriguez-Garcia M. (2020). Qualitative analysis of housing demand using Google trends data. Econ. Res..

[60] Chen T., Wang Y., Yang J., Cong G. (2020). Modeling public opinion reversal process with the considerations of external intervention information and individual internal characteristics. Healthcare.

[61] Lopez V.K., Shetty S., Kouch A.T., Khol M.T., Lako R., Bili A., Ayuen A.D., Jukudu A., Kug A.A., Mayen A.D. (2021). Lessons learned from implementation of a national hotline for Ebola virus disease emergency preparedness in South Sudan. Confl. Health.

[62] Cho Y., Hwang J., Lee D. (2012). Identification of effective opinion leaders in the diffusion of technological innovation: A social network approach. Technol. Forecast. Soc. Chang..

[63] Zhang L., Li H., Chen K. (2020). Effective risk communication for public health emergency: Reflection on the COVID-19 (2019-NCoV) outbreak in Wuhan, China. Healthcare.

[64] Holroyd T.A., Oloko O.K., Salmon D.A., Omer S.B., Limaye R.J. (2020). Communicating recommendations in public health emergencies: The role of public health authorities. Health Secur..

